# Mesenchymal Stem Cell Therapy Regenerates the Native Bone-Tendon Junction after Surgical Repair in a Degenerative Rat Model

**DOI:** 10.1371/journal.pone.0012248

**Published:** 2010-08-18

**Authors:** Geoffroy Nourissat, Amadou Diop, Nathalie Maurel, Colette Salvat, Sylvie Dumont, Audrey Pigenet, Marjolaine Gosset, Xavier Houard, Francis Berenbaum

**Affiliations:** 1 UR 4, Pierre & Marie Curie University, Paris, France; 2 Biomechanics and bone remodeling (EPBRO), Arts et Métiers ParisTech, Paris, France; 3 Department of anatomopathology, AP-HP Saint Antoine hospital, Pierre & Marie Curie University, Paris, France; 4 Department of Rheumatology, AP-HP Saint Antoine hospital, Pierre & Marie Curie University, Paris, France; Hôpital Cochin, France

## Abstract

**Background:**

The enthesis, which attaches the tendon to the bone, naturally disappears with aging, thus limiting joint mobility. Surgery is frequently needed but the clinical outcome is often poor due to the decreased natural healing capacity of the elderly. This study explored the benefits of a treatment based on injecting chondrocyte and mesenchymal stem cells (MSC) in a new rat model of degenerative enthesis repair.

**Methodology:**

The Achilles' tendon was cut and the enthesis destroyed. The damage was repaired by classical surgery without cell injection (group G1, n = 52) and with chondrocyte (group G2, n = 51) or MSC injection (group G3, n = 39). The healing rate was determined macroscopically 15, 30 and 45 days later. The production and organization of a new enthesis was assessed by histological scoring of collagen II immunostaining, glycoaminoglycan production and the presence of columnar chondrocytes. The biomechanical load required to rupture the bone-tendon junction was determined.

**Principal Findings:**

The spontaneous healing rate in the G1 control group was 40%, close to those observed in humans. Cell injection significantly improved healing (69%, p = 0.0028 for G2 and p = 0.006 for G3) and the load-to-failure after 45 days (p<0.05) over controls. A new enthesis was clearly produced in cell-injected G2 and G3 rats, but not in the controls. Only the MSC-injected G3 rats had an organized enthesis with columnar chondrocytes as in a native enthesis 45 days after surgery.

**Conclusions:**

Cell therapy is an efficient procedure for reconstructing degenerative entheses. MSC treatment produced better organ regeneration than chondrocyte treatment. The morphological and biomechanical properties were similar to those of a native enthesis.

## Introduction

Population aging and a better quality of life have led to surgery now being performed on elderly patients who are still very active. The two main orthopedic surgical procedures are joint replacement in cases of osteoarthritis (OA) and the repair of failed tissues. The repair of many joints is particularly challenging in elderly patients because this surgery depends on the natural healing capacity of the body and older people heal more slowly [1]. Tendons and ligaments are fibers made up of dense connective tissue and they are critical for physiological movement and the stability of joints because of their attachment to bone [2]. Injury to these structures can significantly destabilize joints, resulting in the development of degenerative joint diseases, especially in the upper limbs [2]. The most common disorders affect the supraspinatus tendon of the rotator cuff, the Achilles tendon, the flexor tendons of the hand, and the anterior cruciate and medial collateral ligaments of the knee [3], [4]. Almost 60% of people over 60 suffer from a torn shoulder tendon [3], [5]. Current clinical data indicate that 50% of surgically repaired shoulder cuff tears do not heal properly [3], [5], [6]. These poor healing rates are independent of the surgical procedure used [6]–[8] and the poor healing is statistically linked to a negative clinical outcome [6]. Several extrinsic and intrinsic mechanisms have been proposed to explain the development of rotator cuff disease [3], [9]–[11]. One reason for this failure after repair is the age of the surrounding tissues. Fresh, limited lesions heal more rapidly in healthy patients [6]. Unfortunately, rotator cuff disease usually occurs in the elderly, who obviously have aging tissues [9]. Adipose tissue infiltrates the muscles, structural disorders appear in tendon, mineral changes affect the humeral head, and the enthesis disappears [5]. The enthesis is delimited by the region covering the tendon-to-bone insertion [11]. This fibrocartilage consists of four distinct zones: the tendon zone (organizing the anchoring of tendon type I collagen fibers arriving at the enthesis), the unmineralized fibrocartilage, the mineralized fibrocartilage and the bone. The calcification front is the boundary between the two fibrocartilage zones [4]. This four-layer structure is a biomechanical organ transmits loads and decreases the concentration of stress at the attachment zone, acting like a tectonic plate [4]. Many elements are involved in this particular biomechanical function, but a main one is the presence of chondrocytes producing type II collagen [4], [11]. Aging or acute trauma can cause the tendon to become detached from the bone by breakdown or destruction of the enthesis [5], [10], [11].

The high failure rate after repair is independent of the technical procedure used. Hence, orthopedic research has focused on ways to enhance the healing rate [7], [8], [12]–[15]. Both cell therapy and tissue engineering have been investigated [12]–[15]. Several animal studies have explored the use of adjuvants like soft tissue allografts and allogenic growth factors to enhance bone-tendon healing [15]–[18]. One possibility is to use an allogenic scaffold. Cells may be embedded in it to reinforce anchoring or to increase the size of the tendon [17]. Whereas animal studies have reported improvements in load-to-failure, early clinical studies did not reproduce this effect [13], [14], [17]. Another avenue of research is to use recombinant growth factors to promote and enhance the natural ability of the bone to repair the tendon [15]. Experiments using BMP in bone-tendon fixation on sheep showed that it tends to increase load-to-failure in biomechanical tests performed 6 to 12 weeks after repair [15]. Because natural bone-tendon fixation involves a fibrocartilage [4] several authors [19] have studied the effect of implanting cultured chondrocytes between the bone and tendon. But they reported only histological data and no biomechanical tests were performed. One of the main reasons for the paucity of data on cell therapy in bone-tendon healing is the lack of a suitable animal model.

The first aim of this study was to develop and validate a small animal model for exploring the capacity of cell treatment to restore the enthesis. Cell therapy is difficult to use on the rat supraspinatus [20] and there are many data on the biomechanics of the Achilles' tendon and calcaneus of Wistar rats. We therefore developed a model based on the destruction of the enthesis of the Achilles' tendon. The model needed to have a spontaneous healing rate after repair of the bone-tendon junction of 50%, similar to human surgical outcomes. The second part of this study evaluates the effect of depositing chondrocytes or mesenchymal stem cells (MSCs) during the initial repair. Chondrocytes are the natural cells of the enthesis and can produce type II collagen, the major promoter of anchorage. Because the body contains few chondrocytes, we evaluated the capacity of MSCs to produce the same effect as chondrocytes.

## Results

### Experimental model of Achilles' tendon repair

This study was approved by the animal research committee of our university and all animal experiments complied with the guidelines of the Institutional Animal Care and Use Committee.

**Figure 1:**
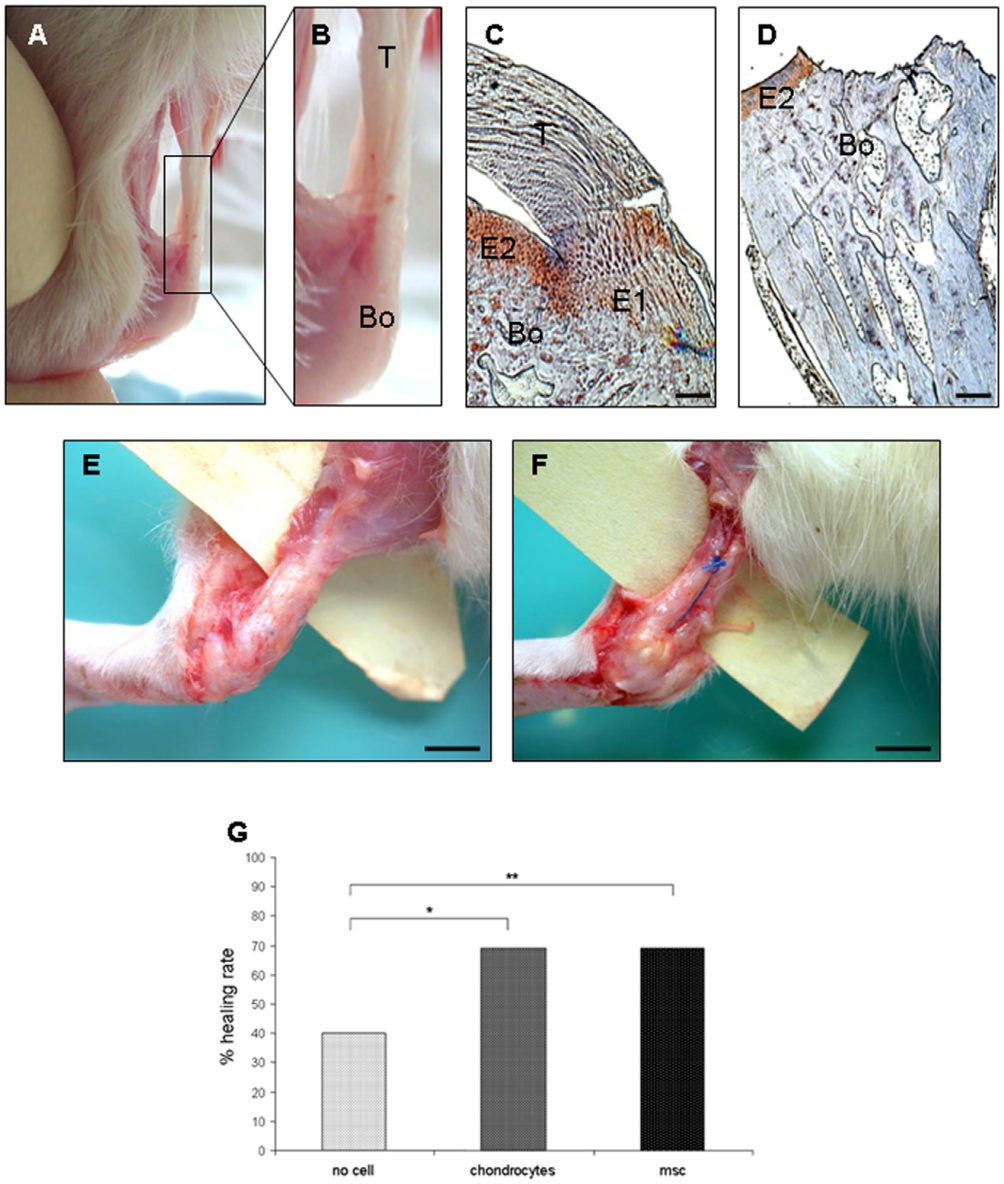
Enthesis structure and healing ratse after repair. The enthesis is the area of bone-tendon junction. A) Wistar rat Achilles tendon-bone junction. B) Higher magnification of the delimited area shown in (A). T, tendon; Bo, bone. C and D) Type II collagen immunostaining of a native (C) and a destroyed enthesis (D). Native enthesis shows two positive areas of collagen II staining: E1, the insertion of the tendon (T) into the bone (Bo) and E2, the sliding zone of the tendon. Note the absence of the tendon and the E1 area just after destruction of the enthesis (D). (C and D, scale  = 200μm). E and F) Healing failure after repair was evaluated by suture breakage (E) and a distance between the bone and the tendon of greater than 1 cm (F) (E and F, scale  = 1 cm). G) Healing rate was evaluated at sacrifice for the three groups of rats and expressed as a percentage. * p<0.05 and ** p 0.01.

The enthesis, the area of bone-tendon junction (Fig. 1A, B), is made up of two areas that stain positively for collagen II. These correspond to the insertion of the tendon into the bone (E1) and the sliding zone of the tendon (E2) (Fig. 1C). The tendon healing model required the destruction of the enthesis followed by the attachment of the tendon to the bone using bony tunnels with sutures (see Material and Methods). The surgical procedure totally destroyed the enthesis, as evidenced by the absence of both tendon and collagen II-positive fibrocartilage (Fig. 1D). In contrast, the E2 cartilage was present after the destruction of the enthesis. Healing failure was evaluated at sacrifice by three macroscopic criteria: 1) the proximal migration of the tendon with a gap between the tendon and the bone, 2) suture rupture (Fig. 1E, F), and 3) the distance between the bone and the muscle of >1 cm. These criteria gave an overall healing rate of 40% (control group G1) (Fig. 1G). The healing rate of our model is therefore similar to that of human shoulder cuff surgery [3].

### Improvement in overall healing rate by chondrocyte and MSC cell therapy

Rat chondrocytes (group G2, n = 51) or MSCs (group G3, n = 39) were injected into the repair site before tightening the suture to determine the effect of cell therapy on the healing rate after enthesis destruction and repair. The overall percentage of healing was significantly higher than in the G1 control group when both chondrocytes and MSCs were injected (Fig. 1G). Only 40% of the tendons healed when no cells were injected (control group G1, n = 52), whereas the healing rate rose to 69% for the two groups of cell-treated rats (p = 0.0028 between G2 and G1, and p = 0.006 between G3 and G1). There was no statistically significant difference between G2 and G3 (p = 0.9). Both cell therapies increased the overall tendon-to-bone healing rate after surgical repair.

### Influence of cell therapy on the load-to-failure 45 days after repair

**Figure 2:**
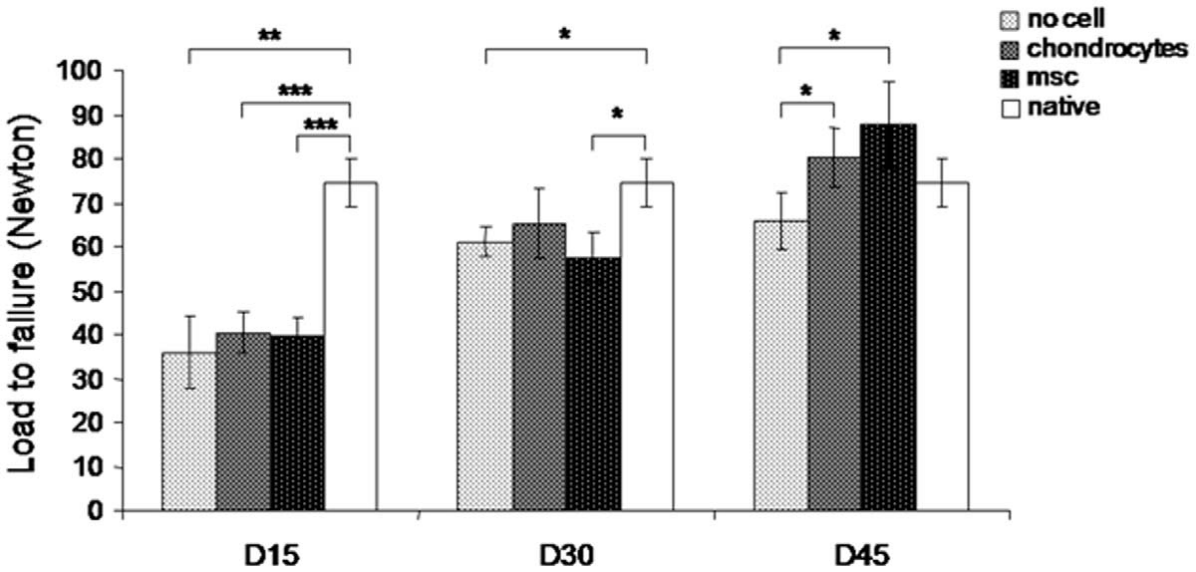
Improvement by cell therapy of the load-to-failure after repair of Achilles tendons. We determined the Load-to-failure of Achilles tendons taken from control (G1), chondrocyte- (G2) and MSC-injected (G3) rats 15, 30 and 45 days after repair and compared them to those of a native enthesis (74.4±10.9 N, white box). There was a statistically significant difference between the value for the native entheses and those for other populations at 15 days post-repair. There was a statistically significant difference between the values for the native enthesis and those of G1 and G3 30 days post-repair, and a statistically significant difference between G1 and G2 and G3, 45 days post-repair. * p<0.05, ** p<0.01 and *** p<0.001.

The load-to-failure is the load required to rupture the bone-tendon junction. Rupture was obtained 15 days after surgery with a significantly lower load in the control G1 group (35.9±13.4N) than in an uninjured enthesis (74.4±10.9 N, p = 0.001) (Fig. 2). The injection of neither chondrocytes (40.5±9.5 N, p<0.001) nor MSCs (39.6±8.7 N, p<0.0001) improved the load-to-failure after 15 days. The values were similar to those for the G1 group and significantly lower than those for the native enthesis (Fig. 2). The load-to-failure values increased with time after surgery in all three groups of rats and reached a plateau between days 30 and 45 for the G1 group. The G2 and G3 rats did not reach this plateau and the load-to-failure values increased further between days 30 and 45. The load-to-failure values 45 days after repair were significantly higher in chondrocyte-injected G2 (80.3±13.0 N) and MSC-injected G3 (84.6±17.1 N) groups than in the G1 rats (68.7±15.1 N) that had been given no cells (Fig. 2). The load-to-failure values for the two cell-injected groups 45 days after repair were also higher than those of native entheses. Thus, cell therapy improved the strength of the repaired tendon.

### Restoration of a native-like enthesis by MSC-injection

**Figure 3:**
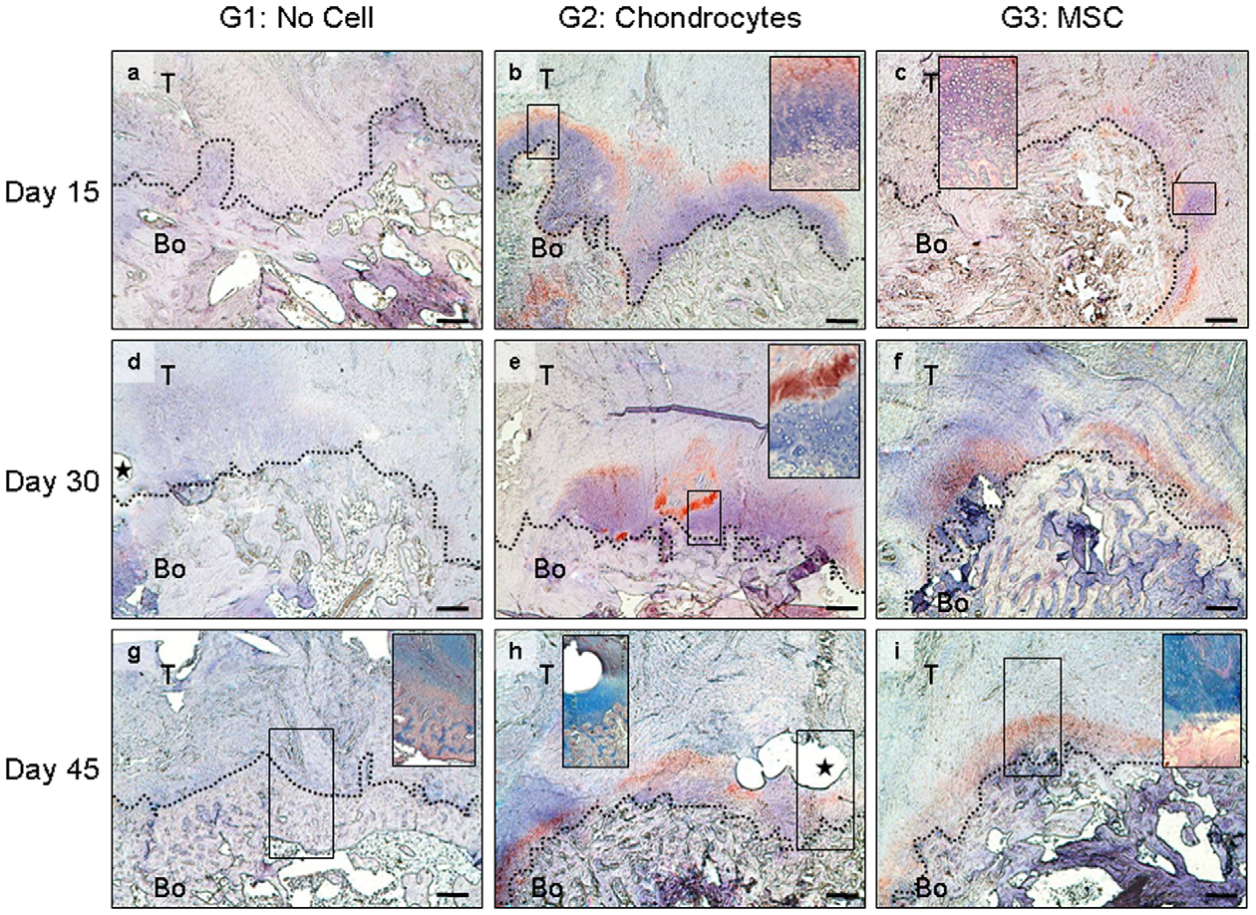
Enthesis production induced by cell therapy after repair. Immunostaining of collagen II in the enthesis of control G1 rats (a, d, g), chondrocyte-injected G2 rats (b, e, h) and MSC-injected G3 rats (c, f, i) 15, 30 and 45 days after repair. There was no collagen II staining of G1 control enthesis on day 15 (a), whereas the bone-tendon junction of cell-injected rats was positively stained (b, c). Chondrocyte- (b) and MSC-treated repairs (c) also contained large aggregates of cells on top of the bone that corresponded to injected cells. (b, c insets) Higher magnification views of delimited areas showing cell aggregates. There was no type II collagen matrix detected in the repairs without cells on day 30 (d). Cell therapy was associated with the deposition of a collagen II matrix, which appears to be more abundant when chondrocytes were injected (e) than when MSCs were used (f). Insert in (e) is a higher magnification view of the delimited area of the corresponding panel, showing cell aggregates close to the collagen II matrix at the bone-tendon junction 30 days post repair. There was neither type II collagen matrix (g) nor GAG (g, inset) in the G1 repairs without cells 45 days post repair. In contrast, a homogeneous collagen II- and GAG-rich matrix covered the bone at the tendon insertion in the cell-injected rat (h, i). Insets (g-i) show alcian blue staining of GAG in the delimited area in the corresponding panel. Bone (Bo) is localized at the base of the image and tendon (T) above it. Tendon and bone areas are delimited by dotted lines. Stars show the hole left by the suture used for repair. Scale  = 200μm.

**Figure 4:**
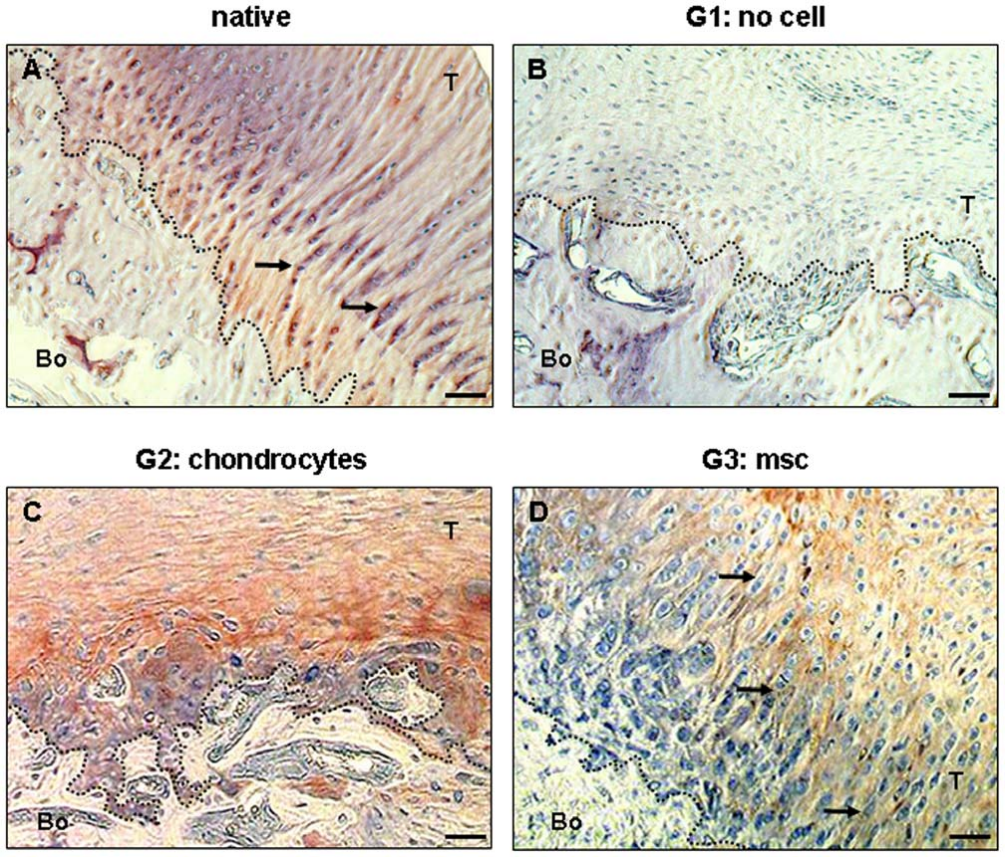
Restoration of enthesis organization by MSC-injection 45 days post repair. Immunohistochemical staining for collagen II was performed on native entheses (A) and 45 days post-repair enthesis from control G1 (B), chondrocyte-injected G2 (C) and MSC-injected G3 rats (D). A) Native enthesis contained columns of chondrocytes (arrows) at the bone-tendon junction and there was positive collagen II immunostaining at the junction. B) Control bone-tendon junction 45 days after surgery showing the absence of both cells and type collagen II. In contrast, the bone-tendon junctions of repairs with cells injected were intensely stained for collagen II (C and D). However, the collagen II distribution was not uniform and there were no chondrocyte columns in chondrocyte-injected rats (C), whereas the repairs of MSC-injected rats 45 days after surgery showed uniform type II collagen distribution at the bone-tendon junction and the chondrocytes formed columns (arrows) (D). Scale  = 200μm. T, tendon; Bo, bone. Dotted lines delimitate bone and tendon.

No collagen II imunostaining was detected in control G1 group of rats 15 and 30 days after surgery and only a faint positive staining was observed on day 45 (Fig. 3a, d and g), although there was fibrous tissue throughout the healing process. Alcian blue staining did not reveal any glycoaminoglycan (GAG) in the G1 repairs 45 days post-surgery (Fig. 3g inset). The histological scale of enthesis production (table 1) indicated that no enthesis was restored when no cells were injected. In contrast, the bone tendon junctions of cell-injected rats showed collagen II deposits as early as 15 days post-repair (Fig. 3b and c). There was collagen II-positive staining around local cell clusters that delimitated the bone-cartilage junction in both G2 and G3 rats (Fig. 3b, c and e insets). The numbers of cells in the clusters decreased over time, whereas type II collagen and GAG production increased (Fig. 3). A second histological analysis was performed at 45 days to assess the morphological organization of the entheses (table 1). The cells at the bone-cartilage junction were organized into columns in the MSC-injected G3 rats (Fig. 4D). Native entheses contained similarly organized chondrocytes (Fig. 4A). In contrast, the enthesis of neither the G2 nor the G1 rats had this columnar chondrocyte distribution at the cartilage-bone junction 45 days after repair (Fig. 4B and C). Quantification of the organization of the enthesis showed statistically significant differences between the native enthesis and G1 (p = 0.008), between the G3 enthesis and G1 (p = 0.027), between the native and G2 entheses (p = 0.005) and between the G3 enthesis and G2 (p = 0.05), whereas the differences between the native and G3, or G1 and G2 entheses were not significantly different. Thus, the histological appearance of the enthesis injected with MSCs at 45 days was similar to that of the native enthesis, but not any of the others (Fig. 4).

**Table 1:** Histological scale used to assess the tissue engineering of the enthesis.

Scoring	Cell at bone-tendon junction	Collagen II at bone-tendon junction	GAG content	Collagen organization	Chondrocyte organization
**1**	No	<25%	No	No	No cells
**2**	Clusters	25 to 50%	Isolated area	Isolated patches around few cells	Isolated cells
**3**	Large areas	50 to 75%	Large areas	Focal area	Clusters
**4**	Over the whole junction	>75%	Over the whole junction	Covering bone-tendon junction	In columns

The first 3 columns are visual grades assigned by examining x 25 images of the bone-tendon junction. The last 2 columns are assessments of x 100 images. The first column uses sections stained with H&E and the second immunostained for type II collagen. The third column sections were stained with Alcian Blue. Columns 1 to 3 were used to define the production of an enthesis. Columns 2 to 5 were used to rank the organization of the neo-enthesis (16-point scale). The native enthesis always scored 16 points in double blind trials (SD = 0).

## Discussion

Repair of tendon-bone insertion is a very common surgical procedure, especially for shoulder cuff disorders or anterior cruciate ligament reconstruction. It is one of the most common procedures in sports medicine [3], [5], [11]. Shoulder cuff surgery results in a scar, but 40 to 80% of interventions fail to repair the problem because no healing occurs [3]. Animal models using sheep, rabbits, dogs and rats have therefore been developed to test adjuvant therapies [21]–[24]. The sheep model developed by Gerber et al. [23] can develop a fatty infiltration of the muscle after tendon release, as in the human shoulder. Rodeo et al. [18] and Gerber et al. [23] both reported that the tendon heals naturally after release, even without sutures, by producing a neo-tendon. This makes it difficult to determine exactly where the initial tendon was located under second-look surgery. Rodeo et al. [18] explored the effect of local growth factors on sheep supraspinatus tendon repair. The tendons were systematically torn with the formation of a neo-tendon between the released tendon and the humerus head. The same problem was reported for a rabbit cuff model [24]. However, without healing criteria, it was not possible to determine whether the healing was induced by the adjuvant therapy or by the natural healing ability of the tendon.

It was necessary to develop an animal model to assess the overall healing rate, the main clinical outcome after surgery. Our rat Achilles tendon model is more similar to human shoulder tendons than is the rat supraspinatus tendon used by Galatz et al. [22]. It is a long tendon that is readily exposed because it lies just under the skin. It is inserted into the bone via an enthesis and attached to a well-identified muscle. It is easy to work on, and many animals can be tested because it is not expensive. The criteria of failure are easy to assess. They are: migration of the tendon proximally with a gap between the tendon and the bone, suture breakage, and a distance between the bone and the muscle of over 1 cm. These criteria are reproducible (with inter- and intra-observer coefficients of correlation of 100%) without any specific training. They also had an overall healing rate of 40% after enthesis destruction, which is close to the human rate, and are convenient for evaluating the effects of adjuvant therapy on healing. This is, to our knowledge, the only animal model that can be used to evaluate the overall healing rate. Reports published to date on bone-tendon healing have only explored the load-to-failure rate [13]–[15], [18], [21]–[24]. It is very important to destroy the enthesis. Murrell studied rats whose Achilles tendon had been severed and reported that there was no biomechanical difference between rats who had undergone surgical repair and those who had not. Natural healing occurred within 15 days, as in other animal models. Similarly, there was no difference in the load-to-failure at 15 days in our animals, but the overall healing rates were different [25]. Most of our tendon-bone insertions failed at the enthesis during mechanical tests. Those that failed at the tendon were not included in the data analyses. We demonstrate the importance of enthesis destruction in our model to obtain a global healing rate close to the human rate.

The challenging for regenerative medicine is to develop models as similar as possible to degenerative age-related changes in organs. One of the most widely accepted models of OA, a typical aging joint disease, is the destabilization of the medial meniscus in young mice [26]. We have used a similar approach by surgically destroying the enthesis.

Gulotta et al. [27] explored the effect of applying bone marrow-derived MSCs in a fibrin glue gel to rat shoulders after supraspinatus repair. They found that there was no difference in the load-to-failure for the group injected with MSCs and the group without cell at 30 days, but they did not explore the effect 15 days later. We found a significant difference in the load-to-failure data at 45 days for the rats without cells and those that had be injected with cells. The values for the rats given cell injections were higher. Thus injecting cells (chondrocytes or MSCs) produces a mechanically stronger insertion than does surgery alone.

**Figure 5:**
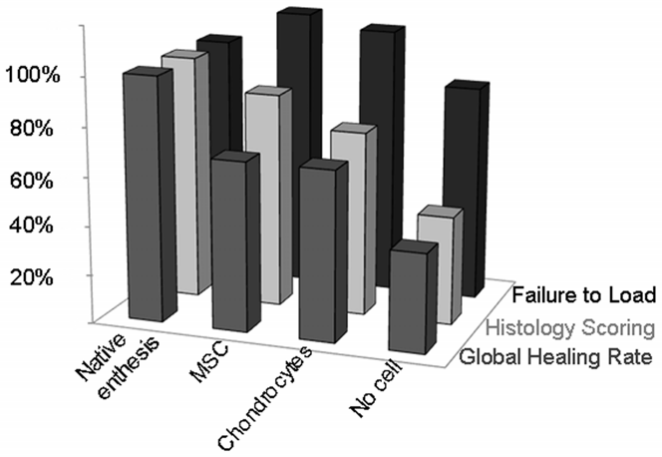
Global results of the study reporting evolution of the histology scoring, failure to load and global healing rate for each population at 45 days. This graph shows the results of each item at 45 days of treatment. The left columns are the data reported for a native enthesis, for animal of the same age as those tested. This graph illustrates the importance of the MSC. While the overall healing rates for the chondrocyte-treated and MSC-treated rats are the same, histological scores and load-to-failure rates of the MSC-treated rats are better than those of the chondrocyte-treated rats.

Histological examination revealed that the local injection of chondrocytes or MSCs stimulated the production of a neo-enthesis at the bone-tendon junction, and that this did not occur after surgery alone. It is not clear whether the chondrocytes at the bone-tendon junctions of the rats given chondrocyte injections were the same as those injected during surgery. They may have come from the calcaneus bone, or from local or general differentiated MSCs. We used chondrocytes to evaluate the effect of cell therapy on bone-tendon healing because they are natural components of the enthesis. They are likely to stimulate bone-tendon healing because they are currently used in clinical practice to produce cartilage [28]. There are reports that chondrocytes can develop the histological appearance of an enthesis when they are placed between a bone and a tendon [19]. Weinand [29] demonstrated that swine chondrocytes placed in an avascular lesion of the meniscus helped the meniscus to heal. But because chondrocytes are rare and difficult to obtain and tend to dedifferentiate in monolayer cultures, we examined the effects of bone marrow-derived MSCs, as they are progenitors of chondrocytes [30]. Gulotta et al. [27] found no difference in the histological production of an enthesis in 2 groups of tendon repairs, one with and the other without MSC injection during repair. They noted the organization of a fibrocartilage with time, but no histomorphometric analysis was performed at 45 days, as the study stopped at 30 days. We found that the MSCs promoted the production of an enthesis at the bone-tendon junction. Stimulated MSCs can produce cartilage and fibrocartilage *in vitro* [31]–[36] but it is not clear just how they act. Gulotta et al. [27] tracked the cells with transduced MSCs and found that they became concentrated in the site of repair, but that there number decreased with time. They may induce the production of a neo-enthesis without any differentiation by releasing paracrine factors that recruit host cells to restore the enthesis. This seems to occur after a myocardial muscle lesion [37]. MSCs may also stimulate the rapid reconstruction of an enthesis. We find that chondrocytes produced type II collagen earlier than did MSCs, and the repaired insertion had a slightly stronger load-to-failure at 30 days than that produced by the MSCs. However, the type II collagen and GAG production in insertions repaired with chondrocytes and MSCs was the same 45 days post-repair. Nevertheless, the structure of entheses induced *in vivo* with MSCs was better (Fig. 4). Histologically, the structure of MSC-induced entheses was very similar to that of native entheses, more so than the chondrocyte-induced entheses. Lastly, repairs made with MSCs gave better load-to-failure values than did repairs made with chondrocytes (Fig. 5), although the difference was not significant. We suggest that the presence (and perhaps better organization) of type II collagen fibers and chondrocytes seen on histology results in greater strength. MSCs seem to be more effective than chondrocytes at regenerating the enthesis so that the tendon is bound strongly to the bone, restoring the histological appearance of the native fibrocartilage.

## Materials and Methods

### Material

All reagents were purchased from Sigma-Aldrich (St Quentin Fallavier, France), unless stated otherwise. Fetal calf serum (FCS) was from Invitrogen (Cergy Pontoise, France). Collagenase D was from Roche Diagnostics (Meylan, France).

### Experimental rat model of degenerative enthesis model repair

All animal experiments complied with the regulations of the Ethical Committee of the Pierre & Marie Curie University animal welfare Department. Approval (P3/2008/003) (Comité Régional d'éthique pour l'expérimentation animale, Ile de France – Paris – Comité 3).

**Figure 6:**
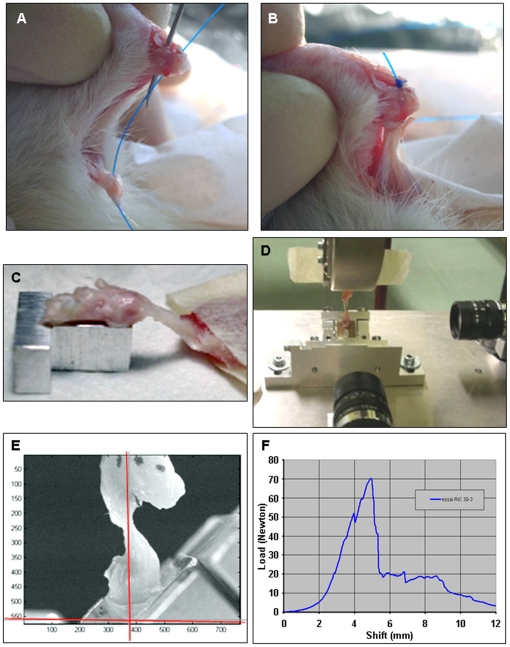
Enthesis repair after destruction and mechanical loading. (A and B) The tendon was retained by a sutures passing through the tendon and two tunnels in the calcaneus bone after destruction of the enthesis. The tunnels were made using a 23G sterile needle (A) and a 4/0 non absorbable suture was passed through the hole to attach the tendon to the bone (B). The plantar side of the calcaneum was placed on a metal rod. The box was fixed to a triangular piece of metal and the fiber part of the muscle was removed, leaving only its aponevrosis fixed in a double-sided tape (C). The proximal tip of the calcaneum was placed on the machine, two CCD cameras were placed at 90° around the sample (frontal and lateral to the calcaneum) to check the positioning (D and E)). A load-to-failure curve was obtained for each sample tested (F).

Male Wistar rats (300 g, 3 months old; supplied by Janvier, Orléans, France) were anesthetized and the Achilles tendon of the left leg was exposed and released from the calcaneum. The tendon was cut at its insertion on the bone, and the enthesis was destroyed with a burr (Fig. 1D). A sterile needle (BD microlance®, 23G) was used to make 2 tunnels through the calcaneum and a 4/0 non-absorbable (4/0 Ethibond) suture was used to attach the tendon back to the bone (Fig. 6A, B). The wound was then closed. The rats were given a pain killer and allowed to recover. They were housed 2 to a cage and allowed to move without restriction. Rats were sacrificed 15, 30 and 45 days after surgery.

A total of 142 rats underwent surgery and were assigned to 1 of 3 experimental groups, as follows: G1, no cells injected (n = 52), G2, chondrocytes injected (n = 51) and G3, MSCs injected (n = 39) at the site of repair just before tightening the suture. Because some rats died and some biomechanical tests or histological assessments failed, surgery was performed to obtain 6 successful biomechanical tests per group and period, and 4 histological assessments per group and recovery time.

### Cell injection

Freshly isolated chondrocytes and expended MSCs (passage 4) were suspended in bovine fibrinogen (40 mg/mL, Sigma) in phosphate-buffered saline (PBS) and mixed with an equal volume of bovine thrombin solution (20 UI/mL, Sigma) [38]–[40] to obtain a final concentration of 4×10^6^ cells/100 μL) [12], [38], [40]. The solution was prepared during surgery, and injected (100 µL) at the site of repair just before tightening the suture.

### Cell preparation and characterization

#### Chondrocyte

Hip and knee cartilages from 4 day-old Wistar rats were harvested as previously described [39]. Briefly, rats were killed by injecting 0.1 mL phenobarbital. The skin on the back and legs was cleaned with antiseptic and removed under sterile conditions. The femurs were dislocated, and the soft tissues around the joints were discarded. Hip and knee cartilages were removed and washed in PBS containing 1% antibiotic. The femoral heads, femoral condyles, and tibial plateaux isolated from two litters (15 rats) were incubated in Dulbecco's Modified Eagle's Medium supplemented with 2 mM L-glutamine, 50 U/mL penicillin and 0.05 mg/mL streptomycin 2 times for 45 min with collagenase D (3 mg/mL) and overnight with collagenase D (0.5 mg/mL) at 37°C under 5% CO2: 95% air. The resulting cell suspension was mixed to disperse cell aggregates, filtered through a sterile 60 μm filter and centrifuged for 10 min at 1500 rpm. The chondrocytes so collected were washed in PBS, suspended in culture medium supplemented with 10% FCS and counted in a hemocytometer.

The average yield per rat was 10^6^ chondrocytes, of which more than 97% excluded Trypan blue. The chondrocytes were injected the day after harvesting to prevent dedifferentiation.

**Figure 7:**
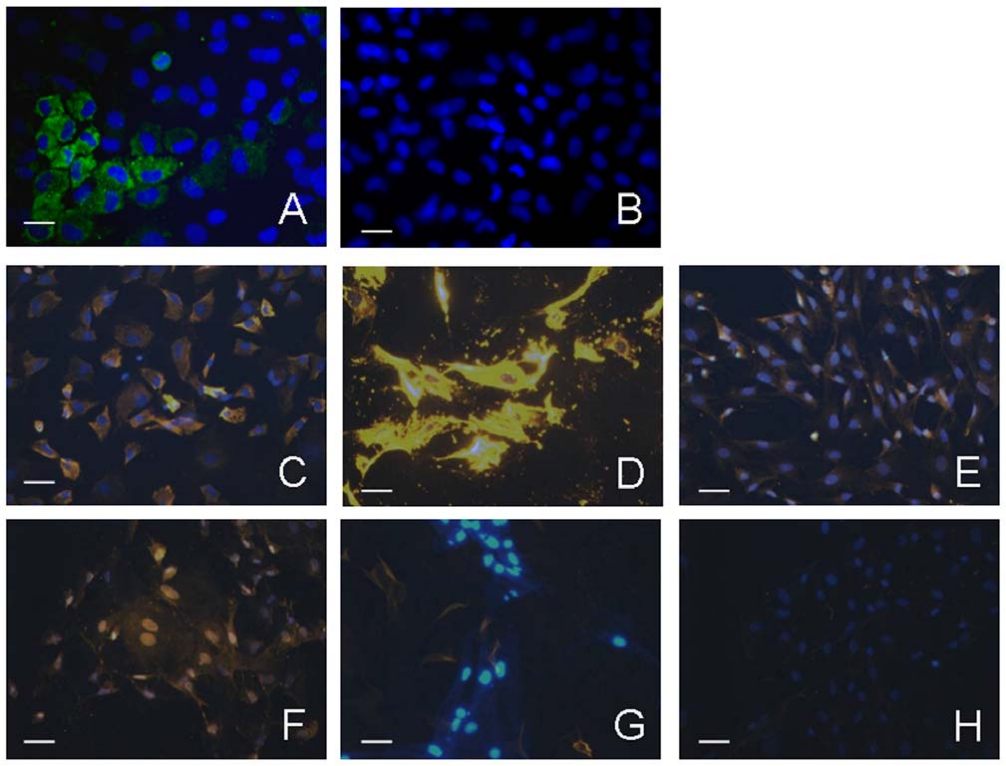
MSC and chondrocyte immuno-characterization. Fluorescent immunostaining. A: type II collagen around chondrocytes and its negative control: B. C: Type I collagen of rat MSCs. D: Fibronectin in rat MSCs. E: Integrin β1 in rat MSCs. F: CD54 in rat MSCs. G: CD45 negative immunostaining of rat MSCs. H: CD14 negative immunostaining of rat MSCs. Scale is 100 µm.

In parallel, cells were seeded on a culture dish (8×10^3^ cells/cm^2^) to confirm their chondrocyte phenotype. At confluence (7 days after seeding), cells showed the typical chondrocyte morphology with a rounded or polygonal shape and granular cytoplasm. The chondrocyte phenotype was confirmed by immunostaining for type II collagen (Chondrex, Redmond, WA, USA) (Fig. 7) and by Alcian Blue staining for sulfated proteoglycans. Alkaline phosphatase staining was performed to demonstrate that there were no bone cells in the primary cultures.

#### Mesenchymal Stem Cells

MSCs were obtained from 4 day-old Wistar rats. The lower limbs were removed as described above and the femur and tibia shafts were immediately placed in a sterile hood and flushed with MSC medium (alpha MEM supplemented with 1% alpha glutamine, 1% antibiotic, 10% Hyclone SVF) containing heparin (5 IU/mL).This solution was passed through a 60 μm filter and centrifuged for 10 min at 1500 rpm. The resulting cells were washed 3 times in PBS containing 1% antibiotic and seeded on 75 cm^2^ plates for 48 h. The medium was then changed to remove dead cells and any non-adhering stem cells. The medium was subsequently changed every other day. After 4 passages, MSCs were injected and their phenotype was confirmed.

Cells were cultured on sterile round cover slips placed in 24-well plates. The confluent cells were fixed with 4% neutral buffered formalin. We used the ChemiconR Mesenchymal Stem Cell Characterization Kit (Chemicon, USA) to demonstrate the presence of markers of rat MSCs (integrin β1, CD54, fibronectin and type I collagen) and the absence of hematopoietic cell surface markers (CD14 and CD45) (Fig. 7).

### Overall healing rate

Healing failure at sacrifice was defined by one of three criteria: 1) the proximal migration of the tendon with a gap between the tendon and the bone, 2) suture rupture (Fig. 1E and F), and 3) a distance of >1 cm between the bone and the muscle. The overall healing rate was assessed in a double blind analysis (by CS and GN) of each tendon. The kappa coefficient of concordance was 1.0, demonstrating no inter-observer difference.

### Mechanical analysis

Samples composed of the calcaneum, tendon and distal part of the muscle were removed as a unit at sacrifice. They were placed in a moist paper and plastic wrap and stored at –20°C. Samples were thawed at room temperature before mechanical testing. Tests were performed under near-physiological conditions [41] by applying force to the tendon positioned at 135° with respect to the plantar side of the calcaneum [42] using a custom made device.

The plantar side of the calcaneum was placed on a 3 mm diameter vertical metal rod placed in a box (13×13×10 mm) (Fig. 6). The distal part of the calcaneum was embedded in dental resin (Lang Dental, Wheeling, IL, USA), leaving at least 3 mm of its proximal part outside the resin (Fig. 6). The box was fixed to a triangular piece of metal so that the plantar side of the calcaneum was at 135° to the axis of traction of the testing machine (model 5565; Instron, Canton, MA, USA).

The triangular base was mounted on an adjustable platform that enabled the tendon to be aligned with the machine axis. The proximal tip of the calcaneum was placed on the machine axis indicated by a needle. Two CCD cameras placed at 90° around the sample (frontal and lateral to the calcaneum) were used to check the positioning (Fig. 6).

The fiber part of the muscle was removed, leaving only its aponevrosis. Double-sided tape was placed on this aponevrosis so as to leave 3 mm of tendon free for testing. The double-sided tape was placed in custom made striated jaws fixed in the machine axis.

The initial length of the sample was defined for a preload of 0.1 N. Samples were then loaded at a traction rate of 10 mm/min until failure. They were tested at room temperature and kept moist throughout the test.

The load-to-failure of the healed bone-tendon insertion in the tensile test was the main parameter measured 15, 30 and 45 days after surgery. We used 6 to 8 samples for the final data analysis at each time for each group (G1, G2, G3).

Reference data were obtained by testing 6 unoperated samples (native enthesis) from 4.5 month-old rats to determine the natural load-to-failure of the bone-tendon insertion of the rat Achilles tendon.

### Histological data

Samples were fixed in 3.7% paraformaldehyde, decalcified and embedded in paraffin. 5 µm sections were cut in the same sagittal plane and immunostained for type II collagen and Alcian Blue staining for GAG, as described [43].

Neo-enthesis production and organization were analyzed using two histological scales. First, neo-enthesis production at 15, 30 and 45 days was assessed by measuring type II collagen production at the bone-tendon junction, the GAG content and cell deposition (magnification ×25) (table 1, first three columns). Second, the organization of the enthesis 45 days after surgery was quantified on 4 samples from each group (magnification ×100). Histological scores (1 to 4) were given for each of the following criteria: 1) the percentage contact between bone and tendon with type II collagen production, 2) type II collagen organization, 3) chondrocyte organization and 4) GAG production (table 1). A total score of less than 5 indicated the absence of an enthesis as in the samples whose enthesis had been destroyed; the histological slides of native enthesis routinely scored 16 (p<0.05).

A blinded analysis was performed by three of the authors. The inter-observer variation of the histological scale was very small, with a very low correlation for each criterion (p = 0.003 to 10^−13^). Student's *t*-test showed non-significant p values between criteria, meaning that one criterion was not responsible for the positive or negative nature of any other. Three criteria were correlated: the percentage contact between the bone and tendon, type II collagen production and cells at the bone tendon junction (p<10^−6^). The percentage of bone-tendon contact was correlated with type II collagen production and the GAG content (p<0.002). These correlations are not surprising as the cells were added to produce a matrix rich in type II collagen and GAG.

### Statistics

Results are expressed as means ± SD. We used a one-tailed Student's *t-*test to compare the load-to-failure data for native, G1, G2 and G3 rats at each time. The overall healing rate was assessed using Pearson's Chi-squared test. Histological data underwent statistical analysis. Intra-observer reproducibility was evaluated with a one-tailed Student's *t*-test on correlation coefficients. We also performed one-tailed Student's *t-*tests to demonstrate the independence of each item of the scale and to compare the histological scores of the groups. Significance was set at p<0.05.
